# Intrinsic Embedded Sensors for Polymeric Mechatronics: Flexure and Force Sensing

**DOI:** 10.3390/s140303861

**Published:** 2014-02-25

**Authors:** Leif P. Jentoft, Aaron M. Dollar, Christopher R. Wagner, Robert D. Howe

**Affiliations:** 1 Harvard School of Engineering and Applied Sciences, Cambridge, MA 02138, USA; E-Mail: howe@seas.harvard.edu; 2 Department of Mechanical Engineering and Materials Science, Yale School of Engineering and Applied Science, New Haven, CT 06511, USA; E-Mail: aaron.dollar@yale.edu; 3 Formerly with the Harvard School of Engineering and Applied Sciences, Cambridge, MA 02138, USA; E-Mail: cwagner@post.harvard.edu

**Keywords:** sensors, embedded, polymer, robotic, shape deposition manufacturing, rapid prototyping

## Abstract

While polymeric fabrication processes, including recent advances in additive manufacturing, have revolutionized manufacturing, little work has been done on effective sensing elements compatible with and embedded within polymeric structures. In this paper, we describe the development and evaluation of two important sensing modalities for embedding in polymeric mechatronic and robotic mechanisms: multi-axis flexure joint angle sensing utilizing IR phototransistors, and a small (12 mm), three-axis force sensing via embedded silicon strain gages with similar performance characteristics as an equally sized metal element based sensor.

## 1. Introduction

In order to become commercially viable, the majority of robotic and mechatronic systems must eventually become compatible with inexpensive, mass-manufacturing processes. In addition to challenges associated with fabricating structures with intrinsic sensors, a number of properties of polymers, such as multi-axial compliance, thermal insulation, and viscoelasticity/creep, make developing useful compatible transducers challenging. However, aspects such as the kinematic configuration of the structure as well as the state of the loading condition must frequently be sensed, even in the presence of those challenging material properties, in order to allow for the desired performance of the robotic or mechatronic system. In this paper, we explore two types of polymeric transducer configurations designed to give satisfactory performance in spite of these properties: a multi-axis flexural joint sensor for compliant structures, and a three-axis load sensor designed to be miniaturizable and incorporated into the structure of small polymeric devices such as surgical instruments.

Fabrication processes such as multi-material molding and insert molding allow for some expansion of the types of systems that can be easily fabricated with modern processes, but have not yet produced fully-integrated sensorized commercial systems with intrinsic transducers. On the scale of small-batch fabrication of research hardware, a popular polymer-based processes is Shape Deposition Manufacturing (SDM) [1,2], which can allow for the fabrication of compliant mechanisms that are very difficult to fabricate with traditional techniques. Complex mechanisms with embedded components can be created as a single part, eliminating the need for fasteners, and reducing the likelihood of damage to fragile components by encasing them within the part structure. To this point, however, SDM structures have been almost purely passive or open-loop mechanisms, devoid of sensing and feedback control [2–5].

In order to expand the utility of polymeric robotics, sensors must be developed that both integrate with and exploit the characteristics of plastics and rubbers [6–9]. Integrating these sensors with the robot structure promises to greatly reduce fabrication costs and complexity, and to increase the robustness of the resulting robotic systems—a crucial feature for commercially-viable systems. In addition to durability and ease of manufacture, polymeric transducers are desirable for MRI-compatibility, and a number of sensor designs have been explored for this application [9–11]. Additionally, considerable progress has been made towards creating soft sensors that stretch like their biological counterparts [12,13].

In this paper, we describe the development and evaluation of two types of sensors that can be utilized to give good sensing performance despite challenging aspects of polymer material properties. The first of these is an infrared phototransistor-based sensor that provides three axes of deflection sensing for compliant flexure joints. Flexures are a frequently-used and simple way of achieving polymeric joints, but are inherently more complex than simple rotational joints and require special sensing to estimate their multi-degree-of-freedom (DOF) state. The second transducer presented is a three-axis stain-gage force sensor. Force sensing is a useful and often necessary capability for a number of applications in which contact with external object must be controlled. However, traditional designs, while accurate and repeatable, are expensive, heavy, and fragile. By embedding strain gages into the structure of the mechanism, we create lightweight, durable, and inexpensive force transducers that are simple to fabricate.

## 2. Phototransistor Flexure Sensing

Flexures are a simple way to add articulation to polymeric mechanisms. They are low-cost, easy to fabricate, frictionless (but not zero stiffness), and robust—even to off-axis loads. Furthermore, they are an easy way to incorporate compliance, and a single flexure can allow deflection around multiple axes, which provides advantages for grasping [3], proprioception, and to detect contact with the environment by the deflection of the joint [14]. Examples of flexure-based joints in robotic systems include the Sprawl series of legged robots [2], the SDM Hand [3], the UB Hand [15], and Compliant Framed Modular Robots [16], among others.

However, while flexures pose many advantages over traditional revolute joints, they are not compatible with standard approaches to measuring joint position, such as potentiometers or encoders. The pose of single-DOF flexure joints has been measured with piezoresistive bend sensors [15], hall-effect sensors [17], and optoelectronic sensors [18], the latter of which we utilize in this paper, but for a multi-DOF sensor. The only literature on measuring the pose of multi-DOF flexure joints (beyond our preliminary design presented in [19]) utilizes strain gauges distributed along the length of a long flexure joint [16] and achieves fairly good performance, albeit with significant mechanical and computational complexity to do so. It is therefore desirable to develop methods to measure the configuration of multi-DOF flexure joints with simpler methods and without needing to instrument the flexure itself.

### 2.1. Design

**Figure 1.**
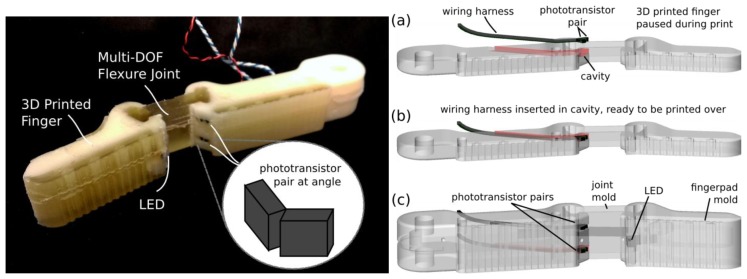
Joint-angle sensor design. An infrared LED shines across the joint onto two angled pairs of phototransistors (**left**). An embedded fused-deposition manufacturing method is used to integrate sensing into the finger design (**right**): (a) print is paused; (b) wired sensor is inserted into cavity designed to hold it; (c) whole assembly is printed over.

The sensor consists of a single infrared LED (VLMD3100-GS08, Vishay Semiconductor, Malvern, PA, USA) shining on to two pairs of phototransistors (four total—OP501DA, Optek Technology, Carrollton, TX, USA), as shown in Figure 1. The two phototransistors in each pair are mounted at different angles, so that as the finger bends, the LED moves from shining directly on one towards shining directly on the other. As the finger twists, the LED moves from one pair to the other, generating approximately 1 V response from each phototransistor (configured as a photodarlington) over a 220-ohm pull-up resistor To calibrate the design, a first-order polynomial approximation is used to map sensor readings to Euler-angle representation of orientation:

$$\left[\begin{array}{c} \theta_{x}\\ \theta_{y}\\ \theta_{z} \end{array}\right]=\left[\begin{array}{ccc} a_{1,1} & \ldots & a_{1,5}\\ a_{2,1} & \ldots & a_{2,5}\\ a_{3,1} & \ldots & a_{3,5} \end{array}\right]\;\left[\begin{array}{c} 1\\ v_{1}\\ v_{2}\\ v_{3}\\ v_{4} \end{array}\right]$$

To fabricate the sensor, a wiring harness is created with the phototransistors and LED. This is laid into the plastic finger which is printed by a fused-deposition manufacturing process (3D printer). After a cavity for the sensor is printed, the printer is paused and the harness is laid inside as shown in Figure 1. Printing then resumes, and as plastic is extruded over the sensor it fixes it in place. This process both provides a cavity to align the sensor, and removes the need for later assembly. The finger design includes cavities for flexure joints (16 mm × 6 mm × 17 mm) and finger pads, which are then filled with two-part urethane rubber (PMC 780, Smooth-On Inc., Easton, PA, USA—Shore-A durometer 80). The walls of the cavities are then peeled off, leaving the flexure joint as shown in Figure 1.

### 2.2. Methods and Results

**Figure 2.**
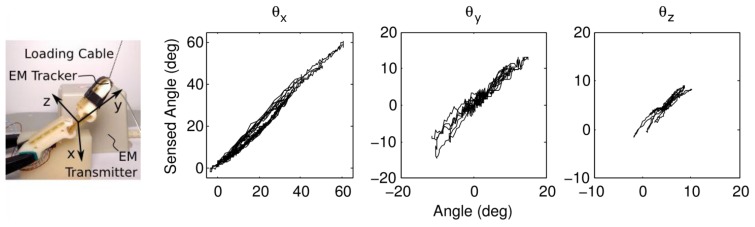
Sensor response: (**left**) experimental setup and (**right**) sensed angles across joint *vs.* actual angles measured with electromagnetic tracker.

To test the response of the finger, the orientation of the distal link is measured with an electromagnetic tracker (TrakSTAR, Ascension Technologies, Shelburne, VT, USA) at 50 Hz; voltages are measured at 10 bit resolution with an Arduino Micro (Arduino, Ivrea, Italy) at 50 Hz, and interpolated in MatLab. The finger is loaded from the tip using a string (simulating fingertip contact), and the results are plotted in Figure 2. The respective performance of the sensor for each degree of freedom is shown in Table 1. Note that the varying stiffness of the joint in different degrees of freedom results in differing magnitudes of deflection.

### 2.3. Discussion

The results show that the design is capable of measuring the deflection of multi-DOF flexure joints, and demonstrate a new method to integrate sensors into polymeric devices. While the errors are higher than seen in typical rotational encoders, there is no other published work measuring 3-DoF flexure deformations to the authors knowledge, and compliant fingers that adapt to the shape of object require less precise information regarding finger placement than do stiff pin-jointed fingers.

The primary source of error comes from the simple calibration between sensor values and flexure deformation (note that only first-order calibration terms are used to avoid overfitting). The flexure is able to deflect in all six degrees of freedom (translation and rotation), but only rotations are measured (the most significant deflection modes—flexion about *x*, *y*, and *z* in Figure 2). However, the flexure is significantly stiffer in translational degrees of freedom due to the joint and finger geometry (the flexure and distal link are roughly ten times longer than the flexure thickness) so these other modes play a less significant role in finger behavior.

Embedding the sensors during the printing process provides a number of advantages. The printed device itself serves as an alignment jig, enabling faster assembly and tighter tolerances. The printed material also provides protection for fragile wires (and, for some sensor types, the sensors themselves).

## 3. Strain-Based Force Sensing

Multi-axis force sensing provides essential information about the interaction between the robot and the environment. Strain gages are often used in high quality force transducers due to their high sensitivity. However, the process of bonding strain gages to the transducer substrate is complicated and time-consuming, and often fails if any of the steps are not performed exactly to specification. Prior work by the authors has found that embedded strain gages can bond well to the structural material without the need for adhesives and give accurate measures of the structural strain in the part [6]. Integrating strain gages into polymeric structures via molding therefore may allow devices to be created with the sensitivity of strain gauges combined with the high durability and ease of construction of polymeric molding. In the following sections we present the design and evaluation of a miniature three-axis force transducer created using the SDM process, consisting of multi-stage molding steps.

### 3.1. Miniature Three-Axis Force Sensor

We integrated six strain gages to create a miniature three-axis force sensor (5 mm × 6.5 mm × 14.5 mm). Our primary motivation for targeting a design of this size was to meet the stringent design requirements of a force-sensing grasper jaw for use with minimally invasive surgical instruments [20,21], and as such, must fit through a small port (5 mm to 12 mm in diameter).

In the following section, we describe the design, construction, and evaluation of our current prototype, designed to form a pair of force-sensing gripper jaws that fit through a 12 mm port. The same technology can be readily applied to generate 5–10 mm pairs of sensors. This prototype is meant as an evaluation platform for an embedded strain-gage based force transducer on a small size scale, and does not incorporate the ideal jaw geometry for surgical grasping tasks.

#### 3.1.1. Sensor Design and Construction

**Figure 3.**
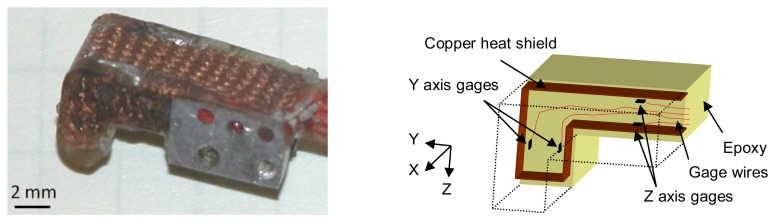
Photograph and cross-sectional diagram of three-axis force sensor. Note copper heat shield.

The force sensor is a dual-serial-beam configuration. One strain gage is located on each of the four sides of the proximal bending beam, sensing two bending moments (corresponding to X and Z forces in Figure 3). Two strain gages are located in the distal bending beam to sense the final axis (Y) of force (Figure 3). The sensor is integrated with an aluminum base allowing simple attachment to manipulators. Because of the SDM process, wires to the individual gages are integrated into the sensor core, removing the need for strain relief. A further advantage is that all wires leave the sensor at the same point providing straightforward and robust wire management.

Silicon strain gages (1 mm × 0.25 mm, SS-037-022-500P, Micron Instruments, Simi Valley, CA, USA) were used to achieve high sensitivity in a small package. Individual strain gage resistances were sensed through a Wheatstone bridge, followed by an instrumentation amplifier circuit with a gain of 100.

The sensor construction is carried out in a two-pour casting process, each with a separate mold cavity. The first pour of liquid polymer embeds the strain gages and wires in an epoxy core (approximately half of the size of the final sensor) and attaches the core to the aluminum base. During this first stage, the gages are suspended in the mold via their lead wires, and positioned by hand to be close to their desired position and orientation. The second pour into a mold of the shape of the final sensor part embeds a copper braid (Ungar-Wick #4, Ungar Products, Apex, North Carolina, CA, USA) heat shield around the core. The epoxy used (Resin 105 Fast Cure, West System, Bay City, MI, USA) was chosen for its stiffness and low creep properties, low mixing viscosity (to fill all parts of the mold), and low curing temperature (to cure inside a wax mold).

A key component of our sensor design is the heat shield to equalize temperature variation between gages. Epoxy is an insulator, which negates the standard temperature differential rejection scheme of examining the difference in strains on opposite sides of a thermally-conducting bending beam. With the heat shield in place, the temperature differences are small and resistance differences between the two gages depends principally on strain.

#### 3.1.2. Sensor Evaluation

**Figure 4.**
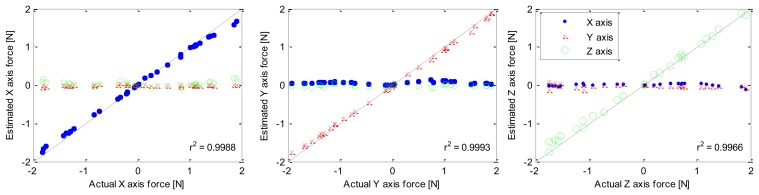
Sensor response to loads showing linearity.

To calibrate the SDM sensor, known loads were applied along each coordinate axis. The calibration matrix was found using a linear least squares method on the gage voltages and known loads. Temperature effects were minimized by allowing the temperature of the sensor to vary during calibration. Estimated forces *versus* known forces are shown in Figure 4 to demonstrate the calibration as well as the linearity (average R2 of 0.998). The force sensor has a range greater than 2 N with an RMS error in calibration of 0.15 N. This range is 40% of the desired full range for a wide range of surgical applications [22], but is appropriate for many tasks.

To characterize noise and resolution, the unloaded sensors were placed in an enclosed container and allowed to thermally equilibrate. Data was then taken for 20 s. Because the major component of noise is at high frequencies, resolution is coupled with sampling frequency. At 1,000 Hz sampling, the RMS noise was approximately 0.1 N.

To examine the benefit of the copper braid heat shield, a sensor was fabricated without the inclusion of a heat shield. After calibration, sensors were allowed to thermally equilibrate in open air. With the non-heat shielded sensor, we measured a drift of 1.2 N over 5 min. The sensor with the heat shield drifts only 0.15 N in 5 min, almost an order of magnitude improvement.

A metal-based force-sensing jaw of the same size scale, using an aluminum element, was also fabricated and is reported in [21]. The resulting average RMS error in calibration was 0.07 N and average R2 was 0.930. Accordingly, the SDM sensor described above has approximately twice the calibration error as the more traditional, metal-based design. However, the ease of fabrication and acceptable performance of the molded sensor make it more appealing for many applications, particularly those requiring “disposable” hardware.

### 3.2. Discussion

Due to thermally-insulating and viscoelastic properties of most polymers, force transducers constructed with polymeric support structures will likely never perform as well as metal-based sensors. The benefits of the SDM approach, however, are many. The force sensitivity of strain gages is retained without the complex bonding process normally associated with strain gages. The sensor is robust due to its monolithic construction. The force ranges can be easily adjusted by depth of gage placement within the element. Wire management is straightforward, with all wires exiting the sensor at the same point, and intrinsic strain relief is provided by the epoxy. Finally, a construction process in which a single pour would encapsulate the strain gages and the heat shield could lead to a straightforward mass production scheme. In mass production the sensors could be low cost and potentially disposable, thus removing the issue of repeated sterilization for surgery applications.

An advantage of traditional metal-based sensors not reflected in the above SDM design is that they are relatively moment insensitive due to moment rejecting flexure design (binocular-shaped cutouts) where applied forces cause the flexure to translate instead of bending [23]. An SDM based sensor could take advantage of this design principle as well, at the cost of a more intricate mold design.

The viscoelasticity of polymeric structures typically causes undesirable creep effects, which would cause a slowly changing force signal under a constant load. We specifically chose a low-creep epoxy to minimize these effects, but we would expect to observe some creep for loads of long duration (hundreds of seconds). Further work will include investigation of other polymers and inclusion of other materials within the polymer to reduce creep, as well as targeting specific applications that are insensitive to creep, such as short time-duration load sensing.

## 4. Conclusions/Outlook

The design approaches and fabrication techniques presented here demonstrate that sophisticated sensors can be readily incorporated into polymeric structures. A central advantage is that the fabrication process can enable the creation of highly effective sensors by embedding inexpensive, prepackaged transducers to create specialized sensing structures. These sensors are part of the robot structure and are created using the same tools and forming techniques as the mechanical structure, requiring minimal additional effort. This also permits optimization of the overall mechanical properties of the system as well as facilitates cable routing. In the joint-angle sensor presented above, phototransistors and LEDs are molded into a finger during a fused-deposition manufacturing printing operation. This approach is readily extensible to other sensors such as hall-effect sensors and allows easy alignment of the sensors to the device.

In the multi-axis force sensor presented above, the strain gauge wiring is molded within the sensor element, providing strain relief and enhanced robustness. This approach can be applied to the entire robot, with all cabling for sensors and actuators molded into the robot structure. While SDM-like processes were used to fabricate the prototypes presented in this paper, a number of established industrial manufacturing processes exist that can enable similar functionality. Multi-shot injection molding is used to create parts such as tool handles with integrated soft grips and parts with integral o-rings and gaskets. Overmolding and insert molding allow for the embedding of prefabricated components and are used to encase electronic components within polymer housings. These processes and others might be used directly or slightly modified in order to mass-produce inexpensive commercial polymeric robotic and mechatronic devices incorporating the sensor designs presented here.

## Figures and Tables

**Table 1.** Joint sensor performance.

*Angle*	*Range*	*Max Error*	*RMS Error*
θ_x_	[−4, 61]	5.2°	1.7°
θ_y_	[−11, 15]	5.0°	1.3°
θ_z_	[−2, 10]	2.0°	0.60°
